# Diet as a Factor Supporting Lung Cancer Treatment—A Systematic Review

**DOI:** 10.3390/nu15061477

**Published:** 2023-03-19

**Authors:** Jacek Polański, Natalia Świątoniowska-Lonc, Sylwia Kołaczyńska, Mariusz Chabowski

**Affiliations:** 1 Department of Internal Medicine, Occupational Diseases, Hypertension, and Clinical Oncology, Wrocław Medical University, 50-556 Wrocław, Poland; 2 Center for Research and Innovation, 4th Military Teaching Hospital, 50-981 Wrocław, Poland; 3 Department of Clinical Oncology, 4th Military Teaching Hospital, 50-981 Wrocław, Poland; 4 Division of Anesthesiological and Surgical Nursing, Department of Nursing and Obstetrics, Faculty of Health Science, Wroclaw Medical University, 51-618 Wroclaw, Poland; 5 Department of Surgery, 4th Military Teaching Hospital, 50-981 Wroclaw, Poland

**Keywords:** diet, nutrition, lung cancer, outcome, treatment

## Abstract

The purpose of this study was to summarize the evidence from epidemiological studies concerning associations between diet and the effectiveness of treatment for lung cancer. For this review, a literature search has been conducted in the EMBASE and PubMed databases, including papers published between 1977 and June 2022. The term “lung cancer” was used in conjunction with “diet”. Footnotes from the selected papers were also analyzed. The present study is in line with the recommendations included in the Preferred Reporting Items for Systematic Reviews and Meta-Analyses (PRISMA) statement. The review included studies involving adults, including randomized controlled trials (RCTs) and cohort and observational studies. In total, 863 papers were found, with duplicates excluded. Ultimately, 20 papers were reviewed. The present systematic review indicates that vitamin A, ascorbic acid (vitamin C), vitamin E, selenium, and zinc—as antioxidants—can strengthen the body’s antioxidant barrier. Furthermore, preoperative immunonutrition may not only improve perioperative nutritional status following induction chemoradiotherapy in lung cancer surgery patients but also reduce the severity of postoperative complications. Similarly, a protein supply may exert a beneficial effect on human health by increasing average body weight and muscle mass. Omega-3 fatty acid content in the diet and the consumption of their main source, fish, may have some regulatory effect on inflammation in patients with lung cancer treated with chemotherapy and radiotherapy. In addition, *n*-3 fatty acids inhibit tumor cell proliferation and may reduce the toxicity of chemotherapy. Increased energy and protein intake are strongly associated with improved quality of life, functional outcomes, hand grip strength, symptoms, and performance in patients with lung cancer. The use of a supportive diet should be the standard of care, alongside pharmaceutical therapy, in treatment for patients with lung cancer.

## 1. Introduction

Lung cancer is currently the most commonly diagnosed malignancy in Poland. It accounts for almost 15% of all cancer diagnoses and nearly 25% of all cancer deaths. A steady growth in new cases is expected over the coming decades [1]. In men, lung cancer incidence and mortality have shown a downward trend for more than a dozen years. In women, both the incidence of breast cancer and its mortality are continuously growing. In spite of the recent developments in the treatment options available, lung cancer is associated with poor survival rates. Even in developed countries, the overall 5-year survival rate is only approx. 22–24% [2,3]. Despite the fact that smoking still accounts for most lung cancer cases, there are growing numbers of lung cancer not related to cigarette smoking. Generally, based on histopathological images, cancer of the lung can be classified into small-cell or non-small-cell carcinoma. [3] The lung cancer stage significantly affects patients’ overall condition, the course of treatment, and prognosis.

Both cancer itself and its treatment have a significant impact on patients’ well-being and daily functioning. The most common lung cancer symptoms include: shortness of breath, chronic cough, and chest pain. Besides the respiratory symptoms, systemic symptoms may be present, including unintentional weight loss, weakness, reduced exercise tolerance, and poor appetite. Nutritional deficiencies are especially common in patients with advanced and metastatic disease [4]. The symptoms depend on the tumor site, disease stage, and treatment type.

Adequate nutrition and targeted nutritional management play a fundamental role at all stages of cancer management, from active treatment (surgery, chemotherapy, radiotherapy) to recovery after treatment completion, to secondary prevention, and to palliative care. Proper nutrition and diet during radiation and chemotherapy help limit their distressing adverse effects and improve patient quality of life (QoL). Surgical and non-surgical cancer treatment places a considerable burden on the body and stimulates catabolism, thus increasing patient demand for protein and energy [5,6]. Moreover, circulating pro-inflammatory cytokines, released by monocytes/macrophages and lymphocytes as an anti-tumor response, contribute to cancer-associated cachexia. These include tumor necrosis factor alpha (TNF-α), interleukins 1 and 6 (IL-1 and IL-6), and interferons alpha and gamma (IFNα and IFNγ) [7,8]. Another group of contributors to cachexia includes circulating catabolic factors produced by the tumor, i.e., a lipid-mobilizing factor (LMF) and a protein-mobilizing factor (PMF) [9]. The third group of causes includes metabolic and hormonal dysfunction. Carbohydrate metabolism disorders involve intensified gluconeogenesis from amino acids and lactates, Cori cycle activity, and glucose metabolism; lower insulin levels; and insulin resistance combined with poorer glucose tolerance. The main protein metabolism disorders include increased muscle metabolism and increased total protein metabolism with the greater synthesis of acute-phase proteins and the reduced biosynthesis of muscle proteins. Changes in lipid metabolism are associated with decreased lipogenesis, increased lipolysis, lower lipoprotein lipase activity, higher glycerol levels, and hyperlipidemia [7,9].

Multiple medications are used to treat cancer cachexia. Benefits have been demonstrated with progestagen, glucocorticoid, and prokinetic treatment. Effective supportive treatment may involve antidepressants, anabolic steroids, non-steroidal anti-inflammatory drugs (NSAIDs), and cannabinoids.

Considering the documented anti-inflammatory effects of certain nutrients in the human body and the findings from previous research, we hypothesized that diet may support treatment in cancer patients. The association between diet and treatment outcomes or prognosis in patients with lung cancer remains uncertain, as research findings to date are equivocal. The purpose of this systematic review is to summarize the evidence from epidemiological studies concerning associations between diet and the effectiveness of treatment for lung cancer.

## 2. Methods

For this review, a literature search in the EMBASE and PubMed databases was performed. It included papers published between 1977 and June 2022. The term “lung cancer” was used in conjunction with “diet”. Footnotes from the selected papers were also analyzed. The present study is in line with the recommendations included in the Preferred Reporting Items for Systematic Reviews and Meta-Analyses (PRISMA) statement [10].

**Figure 1:**
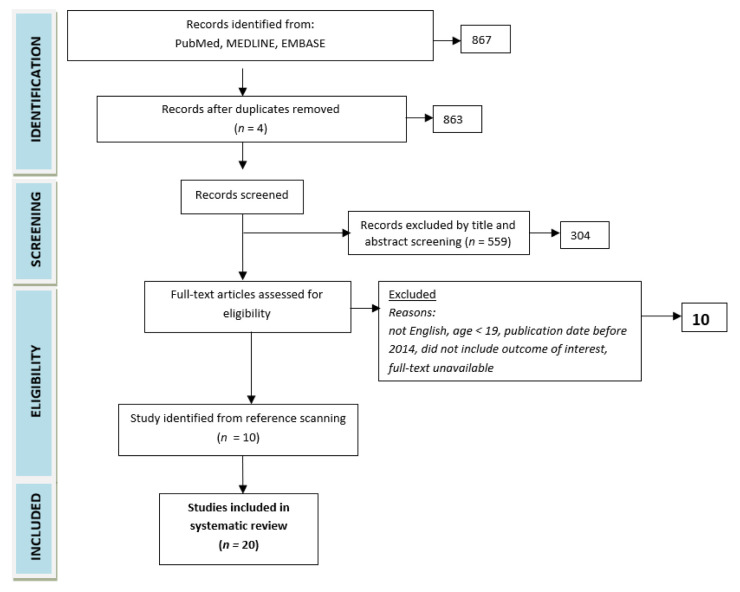
Study flow diagram.

The review included studies involving adults, including RCTs and cohort and observational studies (Figure 1). In total, 863 papers were found, with duplicates excluded. The papers were analyzed and filtered for relevance. A paper was excluded if its findings were not relevant to the subject of the review or if its quality was clearly poor. Ultimately, 10 papers from the initial search and 10 papers cited in the initially identified papers were included in the review. Significant findings or conclusions were extracted from each paper. Evidence for each topic was evaluated using the Bradford Hill [11] and CASP (Critical Appraisal Skills Programme) [12] criteria. Due to the heterogeneity of the data, which is inconsistent with the systematic approach, the evidence has been presented in a narrative review format. A total of 20 studies were analyzed.

## 3. The Role of Nutrients in Cancer Treatment

Nutrients supplied with food provide building materials (proteins, minerals), energy (lipids, carbohydrates, as well as proteins, to a certain extent), and regulating factors (vitamins, fiber, some macro- and micronutrients). Adequately balanced, they support the body in combating cancer. Nutritional strategies that could potentially support cancer management have been extensively studied, but with few conclusive findings.

Like proteins and nucleic acids, lipids are also building materials for living cells and an important energy source, as well as intra- and extracellular signaling molecules [13,14]. Eicosapentaenoic acid (EPA) has been recognized as a fatty acid with specific clinical benefits. Multiple mechanisms explaining the potential benefits of EPA in terms of body composition have been proposed: the inhibition of catabolic stimuli through the modulation of pro-inflammatory cytokine production or increased sensitivity to insulin, which in turn induces protein synthesis. Recent systematic reviews show that EPA may reduce inflammation and potentially modulate nutritional status/body composition [15,16]. In addition, some studies indicate that *n*-3 fatty acids inhibit cancer cell proliferation [17] and may reduce chemotherapy toxicity [18]. Given that a large body of research reports a positive impact of *n*-3 fatty acids on muscle mass, this intervention could be an effective and practical solution helping to prevent muscle loss without any major adverse effects [19]. In a meta-analysis by Thao et al., C Reactive Protein (CRP) and TNFα levels were significantly reduced, which suggests that ω-3 polyunsaturated fatty acids (PUFAs) may have some regulatory effect on inflammation in patients with lung cancer treated with chemotherapy and radiotherapy [20]. Patients suffering from malignant tumors display increased catabolism and resting energy expenditure [21]. Omega-3 PUFAs may affect the latter through the regulation of inflammatory response. Furthermore, they may bind to the cell membranes of muscle fibers and their intracellular organelles, preventing the loss of muscle protein and upregulating its synthesis [22]. Omega-3 PUFAs are considered “immunonutrients”, which are commonly used in nutritional therapy for cancer patients [23]. These fatty acids play a key role in cell membrane fluidity and structure and in cell signaling. Moreover, they are involved in the resolution of inflammation and demonstrate anti-inflammatory effects. Patients with lung cancer experience such complications as pain, anorexia–cachexia syndrome, and depression. The 2017 European Society for Clinical Nutrition and Metabolism (ESPEN) guidelines for cancer patients only describe *n*-3 PUFAs in the context of treating cancer-associated wasting syndrome, without including other cancer-related complications that could potentially be managed with the use of these fatty acids. These fatty acids and their metabolites modulate key pathways underlying lung cancer progression or complications, which constitutes a promising area for research. 

Malnutrition is common in patients with lung cancer undergoing radiation therapy and chemotherapy [24]. Oxidative stress plays an important role in the cytotoxic effects produced by cancer radiation therapy and chemotherapy, as well as in some adverse events. In a study by Tozer et al., 66 patients with stage IIIB–IV NSCLC (non-small cell lung carcinoma) were randomized into a group receiving supplementation with a cysteine-rich protein (IMN1207) or a group receiving casein. Patients receiving the cysteine-rich protein had an average body weight gain of 2.5%, whereas those receiving casein lost 2.6% of body weight (*p* = 0.049) [25]. Moreover, patients with lung cancer who received the supplementation had longer survival, stronger grip, and better QoL. 

The diet of a cancer patient should include more complex carbohydrates and less fast-digesting carbohydrates, which rapidly increase the blood glucose level [26]. 

One of the major side effects of cancer chemotherapy is the increased production of reactive oxygen species, which in turns lead to adverse events that often result in the discontinuation of the treatment [27]. Vitamins A, C, and E as well as selenium and zinc are antioxidants, and may therefore strengthen the body’s antioxidant barrier [28,29,30,31,32,33]. 

Vitamin C inhibits proliferation in lung cancer cell lines [28], triggering cell cycle arrest [29] and apoptosis [30]. Clinical trials [31] have suggested that, when used synergistically with chemotherapy, large intravenous doses of vitamin C may reduce the toxicity or increase the effectiveness of that treatment. A controlled clinical trial by Tokarski et al. showed that after 6 weeks of vitamin C-supplemented chemotherapy, the plasma levels of vitamins A, C, and E significantly increased (*p* < 0.05) in NSCLC patients [32]. The largest increase was found for vitamin C (99.8%). Furthermore, intravenously administered vitamin C may have a positive impact on prognosis in patients suffering from advanced NSCLC [33].

In a prospective, multi-arm randomized study in patients with breast, ovarian, and lung cancer, Mondale et al. investigated the impact of vitamins B12 (methylcobalamin) and E, acetyl-L-carnitine, and glutamine on sensory and motor symptoms and pain from peripheral neuropathy caused by chemotherapy [34]. In the arm treated with 400 mg of vitamin E daily, all symptoms were reduced, and the effect was similar to that of B12 (*p* = 0.446, *p* = 0.227). Vitamin E produced a greater reduction in sensory and motor symptoms and pain compared to both acetyl-L-carnitine (*p* = 0.002) and glutamine (*p* < 0.001).

In experimental carcinogenesis, vitamin A and retinoids have been shown to strongly inhibit the promotion and progression of carcinoma. As suggested by the findings from 26 uncontrolled phase I or II trials included in a meta-analysis by Fritz et al. [35], vitamin A did not have a clear clinical benefit when used independently of chemotherapy. Out of these 26 trials, 13 reported positive outcomes in terms of response rates and/or survival, and 13 reported no significant effect. None of the trials reported disease exacerbation when vitamin A or retinoids had been used in treatment. As reported by Pastroino et al., daily oral administration of high doses of vitamin A effectively reduces the number of new primary tumors associated with tobacco use and may increase the disease-free interval in those patients who had undergone curative resection of stage I lung cancer [36]. 

Selenium and zinc are essential elements with a major role in oxidative stress reduction and DNA protection from reactive oxygen species [37]. Zinc may be protective against tumor initiation and progression. It is also an essential cofactor for several mammalian proteins. Findings show that both these elements effectively support the DNA repair system, which inhibits tumor growth. Piccinini et al. demonstrated that patients with lung cancer presented with significantly lower levels of copper (*p* < 0.01) and zinc (*p* < 0.05) in the hair compared to controls, with no difference in selenium levels. In addition, these patients’ plasma Cu concentrations were higher than in controls [38]. In a study by Tian et al., selenium nanoparticles (nano-SE) combined with radiation therapy showed anti-tumor activity in lung cancer cells through the inhibition of their proliferation, migration, and invasion, as well as the induction of their apoptosis, which suggests that nano-SE may serve as a radiosensitizer in clinical lung cancer treatment, though further studies are necessary [39]. Research findings mainly concern the separate effects of selenium and zinc on various types of cells, tissues, and organs, although their combined effect is for the most part unknown. 

Electrolyte imbalances are a very common complication in cancer patients. They may be associated with poorer treatment outcomes, affecting patients’ QoL, medication options, and survival. Furthermore, recent studies show they may reduce cancer treatment effectiveness; hence, a swift electrolyte level correction may have a positive effect. There is also evidence for an association between electrolyte abnormalities and worse performance status as well as the delays of the treatment initiation and continuation, and adverse outcomes. These abnormalities typically concern the serum levels of sodium, potassium, calcium, and magnesium. Multiple factors may contribute to electrolyte imbalances in cancer patients: the impact of the tumor itself, including the syndrome of inappropriate antidiuretic hormone secretion or tumor lysis syndrome, as well as the impact of cancer treatment and other concurrent clinical conditions or therapies. However, the causes of electrolyte disorders may be complex, and thus it is not always possible to identify and remedy them. 

In a study by Yu et al., a low prediagnostic dietary intake of calcium (<500–600 mg/d) was associated with a slight increase in the risk of death, compared to the recommended intake (800–1200 mg/d). The link between low calcium intake and higher mortality due to lung cancer was mainly found in patients with local/regional tumors (HR (95% CI) 1.15 (1.04, 1.27)). No association between calcium supplementation and survival was found in a model adjusted for multiple variables [40].

Multiple studies have investigated the effect of hyponatremia on cancer patients, indicating a negative correlation with treatment outcomes regardless of its cause [41]. Hyponatremia seems to correlate in particular with worse performance status [42] and reduced survival in patients with lung cancer [43]. The latest evidence indicates that hyponatremia is a significant negative predictive factor in patients undergoing both chemotherapy and targeted therapy [44], whereas a swift correction of this electrolyte disorder improves these patients’ treatment outcomes if the timing allows for avoiding neurological damage [45]. In addition, hyponatremia also appears to negatively affect hospitalized patients, as it has been shown to be associated with a longer duration of hospitalization, poorer QoL and prognosis, and increased cost of hospital stay [46]. Recent evidence also demonstrates that electrolyte channels might be involved in carcinogenesis, emphasizing the importance of electrolyte balance in cancer patients. Multiple potassium channels play a role in cancer proliferation. Potassium channels (KCN) are a large group of proteins involved in potassium conduction. KCNQ1, a pore opening K+ channel, is overexpressed in over 35% of lung tumors, favoring tumor growth, the proliferation and migration of lung tumor cells, and hypoxia resistance [47]. 

According to literature reports, some patients may suffer from cisplatin-induced Mg deficiency [48]. Muraki et al. demonstrated that the administration of fluids with 8 mEq of Mg and mannitol without furosemide prevents cisplatin and pemetrexed-induced nephrotoxicity in patients with advanced NSCLC [49]. In a study by Yoshida et al., preloading 8 mEq of Mg prior to administering cisplatin resulted in a significant reduction in nephrotoxicity caused by cisplatin in the 496 thoracic cancer patients studied [50]. Therefore, Mg supplementation was considered as an option in cisplatin-based chemotherapy. In a study by Oka et al., 85 patients undergoing chemotherapy for lung cancer received fluids supplemented with magnesium or large volumes of fluids without magnesium [51]. In the non-Mg group, serum creatinine levels significantly increased, whereas creatinine clearance significantly decreased compared to baseline. There were no differences in serum creatinine levels or creatinine clearance compared to baseline in patients who received large volumes of fluids with magnesium, whereas those who received small volumes of fluids with magnesium tended to have higher serum creatinine levels and reduced creatinine clearance.

## 4. Diet as a Factor Supporting Lung Cancer Treatment 

Zahra et al. aimed to demonstrate that the ketogenic diet (KD) may be clinically tolerated during radiation therapy and chemotherapy in locally advanced NSCLC and pancreatic cancer and that this might leverage the oxidative metabolism of cancer cells to improve treatment outcomes [52]. Their study included patients histologically diagnosed with pancreatic cancer (AJCC stage IIA, IIB, or III) or NSCLC (inoperable stage III or oligometastatic stage IV). Before the start of the intervention, the patients saw a dietitian to determine their nutritional preferences and caloric demand, sample some foods, and discuss the diet. The KD-compliant meals provided a ratio of 4 g of fat to 1 g of protein and carbohydrates (90% of calories from fat, 8% from protein, and 2% from carbohydrates). Patients followed the KD for approximately 5 to 6 weeks while receiving chemotherapy (gemcitabine 600 mg/m^2^). On average, the patients entered ketosis (≥0.6 mg/dL of beta-hydroxybutyrate) on the third day following the KD, with a continued increase in ketone levels throughout the treatment. The patients consistently showed no decrease in blood glucose levels and no ketoacidosis. Median progression-free survival (PFS) for patients who discontinued the KD early was 7.5 mos. (3.2–33 mos.), whereas the PFS for one known participant who completed the entire course of KD while undergoing radiation therapy and chemotherapy was 4.6 months. In the case of progression, the patients underwent additional therapy, such as stereotactic radiosurgery in the case of brain metastasis or various chemotherapeutic regimens. The median overall survival (OS) in patients undergoing ketolung therapy who discontinued the KD early was 22 mos. (3.7–33.3 mos.), and in those who completed the entire course of KD while undergoing radiation therapy and chemotherapy, it was 17.7 mos. (9.4–26 mos.). The data also show that patients who were in ketosis during therapy had significantly increased carbonyl content in plasma proteins, which suggests that a combination of radiation, chemotherapy, and KD increased the steady-state levels of proteins damaged by oxidative stress. Patients with locally advanced NSCLC undergoing concurrent radiation therapy and chemotherapy had suboptimal oral KD compliance and thus poor tolerance.

Sakoda et al. investigated how common variations in the AGPHD1, CHRNA3, CHRNA5, and CHRNB4 genes are associated with the risk of lung cancer in smokers and how diet can modify that risk [53]. The study included 793 participants of the CARET trial, who provided complete blood samples between February 1994 and February 1997. This was a multicenter randomized double-blind placebo-controlled chemoprevention trial evaluating the safety and effectiveness of daily 30 mg beta-carotene and 25,000 IU retinyl palmitate supplementation for the primary prevention of lung cancer in 18,314 participants found to be at risk. The patients were observed for lung cancer incidence and other endpoints during regular visits to the clinic, phone calls, and mail reports. Food intake in the preceding year was evaluated at baseline and then every two years using the self-administered FFQ questionnaire, specially designed to determine the intake of fruit, vegetables, and specific nutrients. The association between rs16969968 and lung cancer did not differ with the intake of most of the studied nutrients; it was stronger in patients diagnosed at an age <70 years and those with a baseline smoking history of <40 cigarette pack years. These findings suggest that diet has a minor effect on the association between variation in chromosome 15q24-25.1 and the risk of lung cancer.

Lung cancer develops more commonly in the upper rather than the lower lobes. However, its pathophysiological background remains to be explored. Therefore, Lee et al. [54] hypothesized an association between the risk of lung cancer and the consumption of particular vegetables and fruits, and between the route of administration of specific substances (nutrients as well as carcinogens from cigarettes) and the location of the tumor. The study included 328 patients with lung cancer, with the ratio of upper- to lower-lobe tumors being 2.5:1.0. The single-factor analysis included the following predictors of tumor locations: cases of lung cancer in the family, history of smoking and exposure to asbestos, and the consumption of yellow-orange vegetables, α-carotene, β-carotene, and vitamins A, C, and E. The multiple logistic regression analysis included the following independent predictors of upper lobar location: cases of lung cancer in the family (*p* = 0.03), history of exposure to asbestos (*p* = 0.02), lower intake of yellow-orange vegetables (*p* < 0.04), and a lower intake of vitamin E (*p* = 0.05). The findings indicate a strong inverse association between the upper lobar tumor location and the consumption of vitamin E and yellow-orange vegetables.

Jansen et al. investigated the association between the intake of fruits and vegetables and mortality due to lung cancer in a cohort of European men [55]. Complete baseline data were available for 3108 men, including 1578 who smoked at baseline. Over 25 years of observation, 149 of the smokers died of lung cancer. The study found an inverse correlation between the intake of fruits and lung cancer mortality in the participants who smoked (*p* = 0.05). The correlation was only statistically significant in the Dutch cohort ((RR) 1.00, 0.33 (0.16–0.70), and 0.35 (0.16–0.74), *p* = 0.004). In Finnish patients, the risk of lung cancer was reduced, yet not significantly, with higher fruit consumption, and in the Italian cohort, no relationship was found. The consumption of vegetables was not associated with the risk of lung cancer in the smoking participants. Nevertheless, when stratified for smoking intensity, the analyses provided some indication of a lower lung cancer risk in those who consumed more vegetables. In summary, the prospective analysis of male European smokers showed an inverse relationship between fruit consumption and lung cancer mortality. The association was limited and linked to the intensity of smoking.

In a comparative study by Mulder et al. [56], the impact of smoking and diet on lung cancer mortality was examined with the use of aggregated data from the Seven Countries Study [57]. There was a relationship between the 25-year lung cancer mortality specific to the cohorts studied and the prevalence of smoking and the mean dietary intake in the population. There was a positive relationship between smoking and mortality due to lung cancer [OR 1.47, 95% CI: 1.05–2.07]. Furthermore, the study found a positive relationship between lung cancer mortality among smokers and the mean consumption of fats, particularly saturated fats (OR 1.10, 95% CI: 1.04–1.17). However, there was no association with the intake of unsaturated fats, fruits, or vegetables. 

Leedo et al. [58] studied the impact of a home meal delivery service, which offered a range of high-energy and high-protein meals, on QoL in 40 malnourished patients with lung cancer. The patients were randomized to the intervention group (IG), receiving high-energy and high-protein main meals and snacks delivered three times per week, or the control group (CG) continuing with their usual diets. In the IG, there was an improvement in the standard 30 s chair stand test after 6 and 12 weeks (*p* < 0.01) compared with CG. There was a significantly positive impact on performance after 12 weeks (*p* = 0.047). The study found a strong relationship between the increased intake of energy and protein and better QoL, grip strength, as well as score, symptom, functional, and performance results in the studied patients with lung cancer.

A study by Cheng and Neuhouser [59] included 16,693 patients. The researchers found a relationship between the serum concentration of 25(OH)D and lung cancer mortality in non-smokers (HR = 0.53, 95% CI: 0.31–0.92 for former/never smokers and HR = 0.31, 95% CI: 0.13–0.77 for [quit ≥ 20 years]/never smokers). The benefit was smaller in participants with excess levels of vitamin A in the circulation or those taking vitamin A or beta-carotene supplements.

Menezes et al. [60] studied the immunohistochemical expression of nuclear and cytoplasmic vitamin D receptor (VDR) in 63 tumor samples from 35 patients with lung cancer treated at the University of Chicago Hospitals. The authors found that VDR was expressed in numerous samples. In total, 62% of the tumor samples lacked cytoplasmic VDR, whereas nuclear expression was found in 79%. The all-sample analysis demonstrated a positive linear trend between the number of samples with greater nuclear vs. cytoplasmic intensity and increasing histologic grade (*p* < 0.01). The findings suggest that calcitriol may potentially serve as a chemopreventive agent in lung cancer.

In their randomized controlled trial, Sánchez-Lara et al. [61] investigated the impact of an oral nutritional supplement with EPA and an unsupplemented isocaloric diet on clinical, nutritional, and inflammatory parameters and health-related quality of life (HRQoL) in patients with advanced NSCLC. A total of 92 patients with advanced NSCLC received either a diet with an oral EPA-containing supplement (ONS-EPA, *n* = 46) or an isocaloric diet only (C, *n* = 46). All subjects were treated with paclitaxel and cisplatin/carboplatin. The analysis demonstrated significantly higher energy (*p* < 0.001) and protein (*p* < 0.001) intake in the ONS-EPA group than in the control group. In addition, compared to baseline, the ONS-EPA patients gained 1.6 ± 5 kg of lean body mass (LBM), whereas controls lost 2.0 ± 6 kg (*p* = 0.01). There was a reduction in appetite loss, fatigue, and neuropathy in the ONS-EPA group (*p* ≤ 0.05). The groups did not differ in terms of response rate or overall survival.

Arrieta et al. [62] studied the association between malnutrition and serum albumin concentration, on the one hand, and toxicity caused by chemotherapy in NSCLC patients receiving cisplatin and paclitaxel in the other. In the study, 100 patients with stage IV NSCLC receiving paclitaxel (175 mg/m^2^) and cisplatin (60 mg/m^2^) were followed prospectively. In total, 50% of the patients had an albumin concentration of ≤3.0 mg/mL. There was a significant association of a neutrophil–lymphocyte ratio ≥ 5, ECOG = 2 (47.2 vs. 55.4, *p* = 0.026) and a platelet lymphocyte ratio ≥ 150 with baseline body mass index (BMI) ≤ 20 (56.6 vs. 43.5; *p* = 0.02) and hypoalbuminemia (58.9 vs. 41.3; *p* = 0.02). In malnourished and hypoalbuminemic patients, overall chemotherapy-induced toxicity was greater than in those with normal nutritional status (31 vs. 22; *p* = 0.02) and normal albumin levels (mean ranks, 62 vs. 43; *p* = 0.002). There was a relationship between hypoalbuminemia and fatigue (58 vs. 46; *p* = 0.01), anemia (56 vs. 47; *p* = 0.05), and appetite loss (57.1 vs. 46.7; *p* = 0.004) when compared to normal albumin levels. A platelet lymphocyte ratio ≥ 150 was associated with anemia (37.9 vs. 53.8 *p* = 0.004) and grade III/IV toxicity (59.27 vs. 47.03 *p* = 0.008). There was an association of systemic inflammatory response parameters and toxicity with hypoalbuminemia and malnutrition.

Another study by Sánchez-Lara et al. [63] investigated the relationship between nutritional parameters, HRQoL, and survival among patients with advanced NSCLC. The prospective study included 119 patients with advanced NSCLC, not undergoing chemotherapy, and with a good Eastern Cooperative Oncology Group performance status (ECOG 0–2). Bioelectrical impedance analysis with phase angle calculation was performed prior to the initiation of cisplatin-based chemotherapy. The study found an association of malnutrition as measured with the subjective global assessment (SGA), weight loss >10%, and BMI > 20 with lower HRQoL scores. OS was lower in patients with ECOG 2, elevated serum IL-6 concentration, lower phase angle, and malnutrition parameters. Nonetheless, the multivariate analysis showed that the only factors related to poor survival were ECOG 2 (HR = 2.7; 95% CI: 1.5–4.7; *p* = 0.001), SGA (HR = 2.7; 95% CI: 1.31–5.5; *p* = 0.005) and a phase angle ≤ 5.8° (HR = 3.02; 95% CI: 1.2–7.11; *p* = 0.011). The authors demonstrated an association between low HRQoL and malnutrition, the latter being an independent prognostic variable in advanced NSCLC. 

In a randomized controlled trial, Van der Meij et al. [64] analyzed the impact of an oral nutritional supplement with *n*-3 polyunsaturated fatty acids (PUFAs) administered to NSCLC patients undergoing multimodal treatment (concurrent chemoradiotherapy followed by surgery) on the following parameters: QoL, grip strength, performance status, and physical activity. Forty patients with stage III NSCLC undergoing multimodal treatment were randomly assigned to a group receiving two cans of a protein- and energy-rich oral nutritional supplement with *n*-3 PUFAs a day (2.02 g of EPA + 0.92 g of DHA a day) or to a group receiving an isocaloric control supplement. After 5 weeks, QoL, global health status (B = 12.2, *p* = 0.04), physical and cognitive function (B = 11.6 and B = 20.7, *p* < 0.01), and social function (B = 22.1, *p* = 0.04) were significantly higher in the intervention group than in controls. The supplement-receiving group also achieved a higher Karnofsky Performance Status (B = 5.3, *p* = 0.04) compared to controls after 3 weeks. This group also displayed higher physical activity than controls after 3 and 5 weeks (B = 6.6, *p* = 0.04 and B = 2.5, *p* = 0.05). There were no significant differences in grip strength between the groups. In conclusion, *n*-3 PUFAs may have a positive impact on QoL, performance status, and physical activity in NSCLC patients receiving multimodal treatment.

Finocchiaro et al. [65] demonstrated that the administration of EPA and DHA (docosahexaenoic acid) had an anti-inflammatory and anti-oxidative effect in patients with lung cancer. In this multicenter randomized double-blind trial, 33 patients diagnosed with inoperable advanced NSCLC, treated with chemotherapy, were assigned to two groups. Group 1 was administered four daily capsules with 510 mg EPA+ 340 mg DHA (IG), and group 2 was administered 850 mg of placebo (CG), for 66 days. Biochemical (inflammatory and oxidative) and anthropometric parameters were recorded in both groups at the beginning of chemotherapy and after 8, 22, and 66 days. After 66 days of chemotherapy, a significant increase in body weight was found in IG. With regard to inflammation, there were significant differences in CRP and IL-6 concentrations between the groups after 66 days, with the levels gradually decreasing over the course of chemotherapy in IG, demonstrating the anti-inflammatory effect of PUFAs. At later points in the treatment period, the amount of reactive oxygen species in the plasma increased in the CG compared to IG. In the course of the study, HNE concentrations increased in CG and remained stable in IG. 

The clinical trial by Murphy et al. [66] demonstrated that a nutritional intervention involving the administration of fish oil resulted in maintained muscle mass and body weight during chemotherapy. It included 46 patients with NSCLC. From the start to the end of induction chemotherapy, 16 patients received fish oil (2.2 g of EPA daily) and 24 were assigned to the standard of care (SOC). The SOC group lost a mean of 2.3 ± 0.9 kg of body weight, whereas those receiving fish oil maintained their body weight (0.5 ± 1.0 kg, *p* = 0.05). The largest muscle mass increase (r(2) = 0.55; *p* = 0.01) was observed in patients with the largest increase in plasma EPA levels following fish oil supplementation. Muscle mass gain or maintenance was recorded in about 69% of patients from the intervention group. In contrast, there was a 1 kg total muscle loss observed in the SOC group and only 29% of patients in that group maintained their muscle mass. The groups did not differ in terms of total fat tissue.

Another study by Murphy et al. [67] also assessed the impact of fish oil (2.5 of EPA + DHA daily) on clinical benefit and toxicity from chemotherapy and on survival in patients with lung cancer. In total, 31 patients were assigned to the SOC group and 15 to the fish-oil group. The response rate, clinical benefit, and one-year survival were higher in the fish oil group than in SOC (60.0% vs. 38.7%; *p* = 0.15; 60.0% vs. 25.8%, *p* = 0.008; 80.0% vs. 41.9%, *p* = 0.02). There was no difference in a double-lumen tube incidence between the two groups (*p* = 0.46). Compared to SOC, fish oil supplementation increases the effectiveness of chemotherapy with no impact on its toxicity profile. Thus, it may help prolong lung cancer patients’ survival.

A clinical trial by Sun et al. [68] analyzed the effect of selected vegetables (SV) containing known anti-tumor components on survival in patients with stage III–IV NSCLC. The participants were undergoing conventional treatment. They were divided into three groups: TG (toxicity study group) including 5 stage I patients receiving SV in their diet; SVG (intervention group) including 6 stage III or IV patients who also had SV added to their diet; control group including 13 stage III or IV patients whose diet was not modified to include SV. Changes in body weight observed for CG, SVG, and TG were as follows: –12 ± 5%, −2 ± 2%, and +4 ± 4%. The median survival time and mean survival were 4 and 4.8 mos. in the CG, but 15.5 and 15 mos. in the SVG (*p* < 0.01). In the TG, no clinical toxicity symptoms were observed over the 24 mos. of the study. The authors found that the addition of SV to the daily diet of NSCLC patients is not toxic and is related to enhanced body weight maintenance, Karnofsky Performance Status (KPS), and survival in patients with stage III and IV NSCLC.

In patients with lung cancer, poor nutritional intake is a major predictor of perioperative complications. The latter is more likely to occur following preoperative chemoradiotherapy. A comparative study by Shintani et al. [69] investigated how nutritional status in post-chemotherapy patients undergoing lung resection is affected by an immunity-boosting diet. Preoperative nutritional status was compared between 15 patients with lung cancer undergoing lung resection without chemotherapy and 15 who had received chemoradiotherapy. Patients who had undergone chemoradiotherapy had lower BMI and lymphocyte counts. After chemoradiotherapy, six patients received Impact orally (750–1000 mL daily) for five days, and six followed a conventional preoperative diet. The oral administration of Impact for five days prior to surgery modified the postoperative decrease in transferrin levels and lymphocyte counts. The findings confirm that immunonutrition administered before surgery may enhance perioperative nutritional status after first-line chemoradiotherapy in patients undergoing surgery for lung cancer. Such intervention can also contribute to less severe postoperative complications. These potential benefits require confirmation in a randomized controlled trial.

Jagoe et al. [70] performed a detailed nutritional assessment of 52 patients after surgical lung cancer resection. The authors also evaluated the incidence of the postoperative complications, intercostal drainage duration, and the association of preoperative indicators with postoperative results. The authors observed poorer nutritional status, poorer lung function, and lower maximum expiratory pressure in patients who required reventilation or those who died compared to survivors. In multiple regression analysis, the risk of death was predicted by a combination of the type of surgery, preoperative CO transfer factor, and maximum expiratory pressure. The type of surgery and the fat-free mass index alone were only a little less predictive (R^2^ = 0.35). In the case of reventilation, the best model (R^2^ = 0.80) included the type of surgery, BMI, and maximum expiratory pressure (% predicted). Post-lobotomy intercostal drainage duration was only significantly correlated with the preoperative lymphocyte count (*p* = 0.004) and subjective global assessment (*p* = 0.02). Therefore, poor nutritional intake is a vital predictor of death and reventilation following surgical treatment of lung cancer, and patient selection for lung resection should be preceded by the measurement of simple nutritional indicators such as BMI or the fat-free mass index.

Kaya et al. [71] performed an RCT in a group of 58 patients treated with lung resection between January and December 2014. A total of 31 patients were included in a preoperative nutritional program (IG), providing immunomodulating formulas (with *n*-3 fatty acids, arginine, and nucleotides) for 10 days. The control group included 27 patients who received a normal diet. In the IG, 20 patients were treated with anatomic resection by thoracotomy and 11 patients underwent videothoracoscopy. In the CG, 16 patients underwent thoracotomy and 11 underwent videothoracoscopy. Three days after the surgery, a decrease in albumin levels down to 25.71% of the baseline was observed in the CG. In contrast, the decrease was limited to 14.69% in the IG (*p* < 0.001). In total, 12 patients from the CG experienced complications (44.4%) against 6 patients in the IG (*p* = 0.049). The mean chest tube drainage duration was 6 (1–42) days and 4 (2–15) days for the CG and IG, respectively (*p* = 0.019). In summary, the study demonstrated that preoperative nutrition has a beneficial effect in terms of reducing the incidence of complications and chest drainage duration in patients with NSCLC undergoing anatomic resection; in patients who did not receive a nutritional intervention, there was a 25% postoperative decrease in albumin levels.

Shike et al. [57] performed a clinical trial in a group of 31 patients treated with chemotherapy for SCLC to determine the impact of SCLC on body composition, as well as the short- and long-term impacts of parenteral nutrition on body composition and serum protein concentration in patients undergoing radiation therapy and chemotherapy. Subjects were randomized into two groups: the intervention group was administered total parenteral nutrition for 4 weeks, and the control group continued a self-regulated oral diet. There was a significant increase in body weight, total adipose tissue, and total body potassium in the IG over those 4 weeks (*p* < 0.001). No such increase was observed for total body nitrogen. Once parenteral nutrition was discontinued, both body weight and potassium levels in the IG decreased significantly, just as in the CG. Overall, both groups saw a decrease in nitrogen concentrations during the 32 weeks of the study. It was found that the CG experienced a significantly larger adipose tissue reduction compared to the IG once the parenteral nutrition period was over (*p* < 0.05). In other words, parenteral nutrition resulted in increased adipose tissue and total potassium content in the body but did not increase the total nitrogen content in the body (Table 1).

## 5. Conclusions

**Figure 2:**
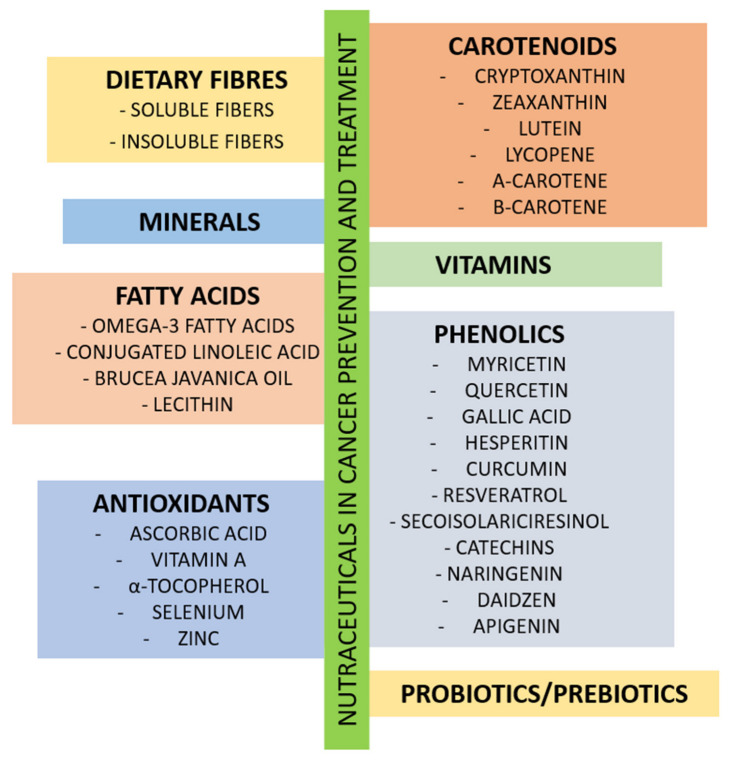
Nutraceuticals in cancer prevention and treatment.

An adequately balanced diet supports the body in combating cancer. Potentially supportive nutritional strategies are a vital part of treatment in patients with lung cancer. The above systematic review of the available data demonstrates that supplementation with essential nutrients and antioxidants may have a beneficial effect on lung cancer treatment (Figure 2).

A proper diet is fundamental to good health and takes on particular importance during illness. Providing nutrients essential to the proper function of the human body is crucial for therapeutic success in patients with lung cancer. The presence of a tumor in the body affects the patient’s nutritional needs, as well as their ability to eat and absorb nutrients. Due to the constant mobilization of the immune system and the long-term nature of the disease, food must provide all essential nutrients, i.e., carbohydrates, proteins, fats, vitamins, and micro- and macronutrients, in the right proportions; otherwise, the body will begin to use up its own tissues for that purpose. Cancer treatment (chemotherapy, radiation therapy, surgery) may also produce a variety of adverse effects and cause patients to forgo certain foods or ingredients, temporarily or permanently. Furthermore, some patients diagnosed with cancer suffer from other chronic conditions associated with various dysfunctions in the body and require specific dietary modifications. Though it will not cure the disease, a well-balanced diet is a very important factor in supporting treatment in cancer patients. Its main purpose is to prevent malnutrition and cancer-associated cachexia. Proper nutrition from the moment of diagnosis can reduce the risk of perioperative complications, minimize the adverse effects of chemotherapy, radiation therapy, or immunotherapy, and strengthen the immune system. Notwithstanding the beneficial influence of dietary interventions on QoL, the data on its value in increasing survival are limited. General information provided by reviewed articles implies that as soon as the disease is diagnosed, nutritional status should be evaluated and an intervention in diet should be administered in order to outrun possible complications, therefore improving QoL, which was proved to be a factor related to survival increase. Overall, any planned intervention should aim at avoiding malnutrition. Data on successful preventive measures with diet against lung cancer are scarce and prevent reliable conclusions, which is a challenge for future research.

This systematic review has several limitations. The primary limitation is the availability and quality of published research articles. Only half of the eligible studies were RCTs or clinical trials. In addition, the vast majority of studies on the effect of diet on the treatment of patients with lung cancer did not meet the quality criteria or lacked the basic information necessary to describe the study. Studies pre-qualifying for a systematic review in terms of quality were few. However, the inclusion of many poor studies may lead to poorly justified conclusions (garbage in garbage out). Furthermore, due to the different study groups, interventions, and study design, the interpretation of such studies is very problematic and may require a meta-analysis or confirmation of the described conclusions in RCTs.

## Figures and Tables

**Table 1:** Description studies included in the review.

No.	Author, Year	Study Design	Study Group	Intervention	Findings and Conclusions
1	Zahra et al., 2017 [52]	Clinical trial, phase 1 (ketolung)	7 patients diagnosed with NSCLC (inoperable stage III or oligometastatic stage IV)	combination of standard radiation therapy and chemotherapy with ketogenic diet (duration: 6 wks.)	Mean actual duration of ketogenic diet use was 16.9 days (0–42 d) of the planned 42 d. Median PFS for patients who discontinued the diet early was 7.5 mos. and median OS was 22 mos. (3.7–33.3 mos.). For one known participant who completed the entire dietary intervention, median PFS was 4.6 mos, and median OS was 17.7 mos. (9.4–26 mos.).
2	Sakoda et al., 2011 [53]	Nested case–control	IG: 746 patients with lung cancer CG: 1477 participants	n/a	No relationship between diet and lung cancer risk among smokers with the 15q24-25 chromosome.
3	Lee et al., 1998 [54]	Case-control	328 patients with lung cancer Lower-lobe tumors: 93 patients, mean age 67.1 ± 9.7 years Upper-lobe tumors: 235 patients, mean age 66.1 ± 9.7 years	n/a	Upper-lobe tumors were significantly more common in patients who consumed less vitamin E (*p* = 0.05) and yellow-orange vegetables (*p* = 0.04).
4	Jansen et al., 2001 [55]	Prospective follow-up cohort study	3108 men from three European countries: non-smokers (never smoked or quit) + smokers eating fruit and vegetables, depending on culture and location Finland: non-smokers *n* = 651 smokers *n* = 637 Netherlands: non-smokers *n* = 288 smokers *n* = 325 Italy: non-smokers *n* = 591 smokers *n* = 616	n/a	Over 25 years, lung cancer mortality was the highest in the Netherlands, moderate in Finland, and the lowest in Italy. Fruit intake was inversely proportional to mortality due to lung cancer in smokers (especially in the Dutch population). Vegetable consumption has no impact on lung cancer risk and mortality in smokers.
5	Mulder et al., 2000 [56]	Comparative study	12,763 men from 7 countries and 16 cohorts, aged 40–59 years (Finland, Italy, Greece, former Yugoslavia, Japan, Netherlands, USA)	n/a	Consumption of saturated fat significantly increased lung cancer mortality in smokers (HR 1.10, 95% CI: 1.04–1.17 for an increase of 4.6 g).
6	Leedo et al., 2017 [58]	Randomized controlled clinical trial	40 patients with lung cancer, with an NRS-2002 score of ≥3 IG (*n* = 21): protein- and energy-rich diet CG (*n* = 19): usual diet	Primary endpoint—QoL after 6 and 12 weeks, follow-up after 3 and 6 mos. Secondary endpoints—performance status, functional score, depression, symptoms, lower body strength, grip strength, body weight after 6 and 12 weeks, follow-up after 3 and 6 mos.	Increased supply of energy and protein in patients with lung cancer shows a tendency towards QoL improvement but does not produce a statistically significant effect; it may improve lower body strength and performance status.
7	Cheng et al., 2012 [59]	Non-human subjects research	Data of 16 693 male and female patients from the NHANES III study, 1988–1994. 258 subjects, 104—former smokers, 23—nonsmokers (data from the National Death Index database)	n/a	In non-smokers, 25(OH)D levels were inversely proportional to mortality due to lung cancer. Benefits were smaller in patients with abnormally high vitamin A intake or blood levels of vitamin A/beta-carotene.
8	Menezes et al., 2008 [60]	Non-human subjects research	Immunohistochemical expression of VDR in 180 precancerous or cancerous bronchial biopsies from bronchoscopy of 78 high-risk subjects, 63 tumor biopsies from 35 patients with lung cancer	n/a	No cytoplasmic VDR found in 38/61 (62%) of tumor biopsies, with nuclear expression observed in 49/62 (79%). All-sample analysis showed a positive linear trend, comparing samples with higher nuclear VDR expression and higher histologic grade (*p* < 0.01). The findings suggest a potential chemoprotective effect of calcitriol on the course of lung cancer.
9	Sánchez-Lara et al., 2014 [61]	Randomized clinical trial	92 patients with advanced NSCLC: IG: 46 (ONS-EPA) CG: 46 (normocaloric diet)	Comparison of an isocaloric diet and diet enriched with ONS-EPA in patients undergoing paclitaxel + cisplatin/carboplatin chemotherapy Response to chemotherapy and overall survival was measured.	IG diet provided much more protein and calories than CG diet. IG gained 1.65 kg of lean body weight; CG lost 2.06. Symptoms, i.e., fatigue, appetite loss, and neuropathy decreased in IG (*p* ≤ 0.05). The groups did not differ in terms of overall survival.
10	Arrieta et al., 2010 [62]	Prospective study	100 patients with CS-IV NSCLC receiving paclitaxel (175 mg/m^2^) and cisplatin (80 mg/m^2^) chemotherapy (mean age 58 ± 10 years). SGA was used to determine nutritional status before treatment.	n/a	Malnutrition in 51% of patients, albumin concentration of ≤3.0 mg/mL in 50%. Neutrophil-lymphocyte ratio ≥ 5 associated with hypoalbuminemia (mean ranks, 55.7 vs. 39 *p* = 0.006), ECOG = 2 (47.2 vs. 55.4 *p* = 0.026), and platelet-lymphocyte ratio ≥ 150 significantly correlated with BMI ≤20 (56.6 vs. 43.5; *p* = 0.02) and hypoalbuminemia (58.9 vs. 41.3; *p* = 0.02). Malnourished and hypoalbuminemic patients had a higher likelihood of developing symptoms of chemotherapy toxicity than normally nourished patients (31 vs. 22; *p* = 0.02) and those with normal albumin levels (mean ranks, 62 vs. 43; *p* = 0.002). Chemotherapy induces more adverse effects in malnourished and hypoalbuminemic NSCLC patients.
11	Sánchez-Lara et al., 2012 [63]	Prospective study	119 patients; 55 female, 64 male (median age 60.5 ± 12.5 y/o, mean BMI 24.8 ± 4.5 kg/m^2^) (A) 40.3% normally nourished per the SGA (B) 32.8% at risk of malnutrition (C) 26.9% severely malnourished Albumin levels were 1.7–4.5 mg/dL, mean 3.3 ± 0.5 mg/dL. Median NLR and PLR 4.7 ± 4.6 and 231.2 ± 162.3, respectively, median CRP level 3.9 mg/dL. Serum proinflammatory factor levels 18.4 ± 31.7 and 21.16 ± 6.4 pg/mL, respectively, for TNF and IL-6.	n/a	Measurement of pre-treatment nutritional parameters contributes to prognosis in patients with lung cancer; this may be a first step towards the unification of studies on the introduction of interventions to improve HRQoL and overall survival. In summary, malnutrition results in poorer HRQoL, the latter being an independent prognostic variable in advanced NSCLC.
12	Van der Meij et al., 2012 [64]	Randomized clinical trial	IG/CG (20/20): 40 patients with CS-III NCSLC	IG: 2 cans/d of calorie- and protein-rich oral nutritional supplements containing *n*-3 PUFAs CG: isocaloric nutritional supplements.	IG had significantly higher QoL, physical and cognitive function parameters (*p* < 0.01), overall health (*p* = 0.04), and social functioning (*p* = 0.04) than CG after 5 weeks. Higher KPS (*p* = 0.04) observed in IG than CG after 3 weeks. No significant differences in grip strength between the groups. IG patients were more physically active than CG patients after 3 and 5 weeks (*p* = 0.05). In summary, *n*-3 PUFAs may positively influence QoL, functioning, and physical activity in NSCLC patients receiving multimodal cancer treatment.
13	Finocchiaro et al., 2012 [65]	Randomized double-blind clinical trial	33 patients aged 46–70 IG: 13 patients out of 19 who completed the trial, aged 46–66 (mean 55.56, SD 7.35 yrs.), took a supplement rich in *n*-3 fatty acids CG: 14 patients aged 50–70 (mean 60.57, SD 7.43 years)	Clinical condition, inflammation, and oxidative stress levels compared over 66 days of observation, until the end of chemotherapy.	Body weight increased significantly in IG patients, who also had reduced systemic inflammatory response and oxidative stress.
14	Murphy et al., 2011 [66]	Clinical trial	40 patients with advanced NSCLC, from diagnosis to completion of 1st line chemotherapy.	IG (*n* = 16) supplemented with fish oil at 2.2 g EPA/d CG (*n* = 24) standard of care Muscle and fat tissue content was compared using computed tomography. Data on blood counts and body weight were collected throughout the treatment period.	In the CG, mean body weight loss was 2.3 ± 0.9 kg, compared to 0.5 ± 1.0 kg in the IG (*p* = 0.05). Patients with a greater increase in blood EPA levels in the IG achieved greater benefits in terms of muscle mass (*p* = 0.01). Muscle mass gain or maintenance recorded in about 69% of patients from IG. In total, 1 kg total muscle loss observed in the SOC group, and only 29% of patients in that group maintained their muscle mass. No statistically significant change in fat tissue was found in either group. In summary, the nutritional intervention with 2.2 g of EPA daily seems superior to standard care, resulting in the maintenance of body weight and muscle mass during chemotherapy.
15	Murphy et al., 2011b [67]	Clinical trial	46 patients with advanced NSCLC	IG (*n* = 15) supplemented with fish oil at 2.5 g EPA and DHA/d during chemotherapy (carboplatin + vinorelbine or gemcitabine) CG (*n* = 31) standard of care	Greater clinical benefit in IG than in CG (60.0% vs. 25.8%, *p* = 0.008; 80.0% vs, 41.9%, *p* = 0.02) No difference in DLT incidence between the two groups (*p* = 0.46). One-year survival seemed higher in IG (60.0% vs. 38.7%, *p* = 0.15). In summary, supplementation with EPA/DHA-rich fish oil is associated with increased effectiveness of chemotherapy, with no impact on toxicity profile, and may contribute to overall survival.
16	Sun et al., 1999 [68]	Clinical trial	CG: 5 patients stage IV, 4 stage IIIB, 4 stage IIIA (54.3 ± 8.8). SVG: 6 patients: 2 stage IV, 3 stage IIIB, 1 stage IIIA (49.2 ± 4.7).	Vegetables with anti-tumor components (SV) were added to the daily diet of TG and SVG, and not added to the diet of CG.	KPS decreased in CG patients but increased in SVG patients within 1–3 mos. of inclusion in the study. Changes in body weight observed for CG, SVG, and TG were as follows: –12 ± 5%, –2 ± 2%, and +4 ± 4%. The median survival time and mean survival were 4 and 4.8 mos. in the CG but 15.5 and 15 mos. in the SVG (*p* < 0.01). The authors found that the addition of SV to the daily diet of NSCLC patients is not toxic and is related to enhanced body weight maintenance, KPS, and survival in patients with stage III and IV NSCLC.
17	Shintani et al., 2012 [69]	Comparative study	IG: 15 patients (aged 62 ± 8 years) with resectable clinical N2 or N3 NSCLC CG: 15 patients (aged 62 ± 6 years) with resectable clinical N2 or N3 NSCLC	IG: received Impact orally (750–1000 mL daily) for 5 days prior to surgery; amount was specified by dietitians based on each patient’s diet during this period. CG: conventional diet before surgery.	Patients undergoing chemoradiotherapy had lower lymphocyte counts, lower BMI, and a higher risk of serious postoperative complications compared to those not undergoing chemoradiotherapy. After chemoradiotherapy, the postoperative decrease in transferrin levels and lymphocyte counts was reduced in the IG. Immunonutrition administered before surgery may enhance perioperative nutritional status after first-line chemoradiotherapy in patients undergoing surgery for lung cancer. Such intervention can also contribute to less severe postoperative complications.
18	Jagoe et al., 2001 [70]	Prospective study	52 patients undergoing surgical lung cancer resection in the years 1995–1997.	n/a	Impaired respiration was associated with nutritional status, poorer lung function, and lower maximum expiratory pressure.
19	Kaya et al., 2016 [71]	Randomized clinical trial	58 patients treated with lung resection	IG: *n* = 31; immunomodulating formulas (with *n*-3 fatty acids, arginine and nucleotides) for 10 days; *n* = 20—anatomic resection by thoracotomy; *n* = 11—videothoracoscopy CG: *n* = 27; normal diet; *n* = 16—thoracotomy; *n* = 11—videothoracoscopy	Three days after the surgery, a decrease in albumin levels down to 25.71% of the baseline was observed in the CG. In contrast, the decrease was limited to 14.69% in the IG (*p* < 0.001). In total, 12 patients from the CG experienced complications (44.4%) against 6 patients in the IG (*p* = 0.049). The mean chest tube drainage duration was 6 (1–42) days and 4 (2–15) days for the CG and IG, respectively (*p* = 0.019).
20	Shike et al., 1984 [57]	Clinical trial	31 patients undergoing chemotherapy for SCLC: IG: 15 patients aged 57.5 ± 2.2 years CG: 16 patients aged 60.4 ± 2.05 years	Total parenteral nutrition for 4 weeks (IG) or continued self-regulated oral diet (CG).	Significant increase in body weight, total adipose tissue, and total body potassium in the IG over those 4 weeks (*p* < 0.001). No such increase was observed for total body nitrogen. Once parenteral nutrition was discontinued, both body weight and potassium levels in the IG decreased significantly, just as in the CG. Overall, both groups saw a decrease in nitrogen concentrations during the 32 weeks of study. CG experienced a significantly larger adipose tissue reduction compared to the IG once the parenteral nutrition period was over (*p* < 0.05).

OS—overall survival; HRQoL—health-related quality of life; VDR—vitamin D receptor; PFS—progression-free survival; QoL—quality of life; ECOG—Eastern Cooperative Oncology Group performance status; HR—hazard ratio; CI—confidence interval; NRS—nutritional risk score; SD—standard deviation; PLR—platelet lymphocyte ratio; CRP—C-reactive protein; NLR—neutrophil-to-lymphocyte ratio; TNF—tumor necrosis factor; IL-6—interleukin 6; KPS—Karnofsky Performance Status; PUFAs—ω-3 polyunsaturated fatty acids; EPA—Eicosapentaenoic acid; SVG—selected vegetables group; TG—toxicity study group; DHA—docosahexaenoic acid; SOC—standard of care; SCLC—small cell lung cancer; NSCLC—non-small-cell lung carcinoma; CG—control group; IG–intervention group; SGA—subjective global assessment; BMI—body mass index.

## Data Availability

The data that support the findings of this study are available from the corresponding author upon reasonable request.
